# An Efficient Method for the Production of High-Purity Bioinspired Large Unilamellar Vesicles

**DOI:** 10.1021/acssynbio.3c00540

**Published:** 2024-02-29

**Authors:** Meline Macher, Amelie Obermeier, Sebastian Fabritz, Massimo Kube, Hannah Kempf, Hendrik Dietz, Ilia Platzman, Joachim P. Spatz

**Affiliations:** † Max Planck Institute for Medical Research, Jahnstraße 29, Heidelberg 69121, Germany; ‡ Max Planck School Matter to Life, Jahnstraße 29, Heidelberg 69121, Germany; § Institute of Molecular Systems Engineering and Advanced Materials, Im Neuenheimer Feld 225, Heidelberg 69120, Germany; ∥ Technical University of Munich, Am Coulombwall 4a, Garching 85748, Germany

**Keywords:** large unilamellar vesicles (LUVs), bottom-up synthetic biology, extracellular vesicles, cellular organelles, liposomes, lipid vesicles

## Abstract

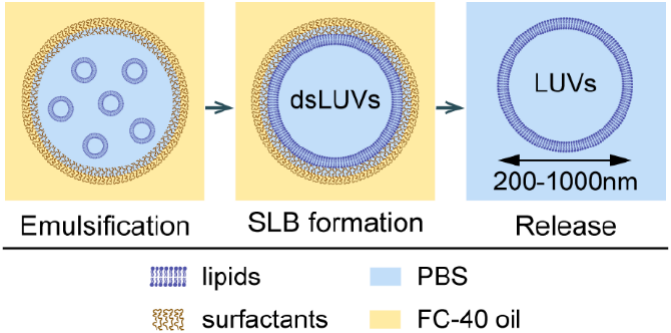

In order to recapitulate complex eukaryotic compartmentalization, synthetic biology aims to recreate cellular membrane-lined compartments from the bottom-up. Many important cellular organelles and cell-produced extracellular vesicles are in the size range of several hundreds of nanometers. Although attaining a fundamental characterization and mimicry of their cellular functions is a compelling goal, the lack of methods for controlled vesicle formation in this size range has hindered full understanding. Here, we show the optimization of a simple and efficient protocol for the production of large unilamellar vesicles (LUVs) with a median diameter in the range of 450–550 nm with high purity. Importantly, we rely on commercial reagents and common laboratory equipment. We thoroughly characterize the influence of different experimental parameters on the concentration and size of the resulting vesicles and assess changes in their lipid composition and surface charge. We provide guidance for researchers to optimize LUV production further to suit specific applications.

## Introduction

Synthetic biology aims to artificially recreate life-like systems, such as cells, cell organelles, synthetic viruses, and extracellular vesicles. These biological systems span different length scales. Whereas the dimensions of prokaryotic cells are in the range of a few micrometers to smaller than 1 μm, the size range of eukaryotic cells is between a few hundred micrometers and 10 μm in size, depending on the cell type and cell state. Eukaryotic cells achieve structural complexity and functional compartmentalization by various endomembrane systems. Most of the intracellular membrane-bound organelles, such as endosomes, peroxisomes, mitochondria, and lysosomes, are usually hundreds of nanometers in size.^1−5^ For some organelles, it is known in what way size is essential for correct functioning, such as in the case of COPII-coated vesicles that have a diameter larger than 300 nm to transport collagen fibers.^6^ Membrane-enclosed compartments are also released into the extracellular space as vehicles for intercellular communication and regulation, termed extracellular vesicles (EVs). EVs can be divided into large (200–1000 nm in diameter) and small (40–200 nm in diameter) vesicles.^7^ One of the central goals of bottom-up synthetic biology is the development of bioinspired cellular compartments to serve as minimal bioactive modules, whose functions can be studied in isolation or integrated into a synthetic cell, natural cell, or even tissue.^8−12^ A crucial prerequisite for this is the ability to assemble lipid membranous compartments in nanometer and micrometer size ranges in a controlled manner.

To cover different sizes of lipid-based compartments, synthetic biology utilizes different approaches to generate giant, large, and small unilamellar vesicles (GUVs, LUVs, and SUVs, respectively). GUVs are often employed to recapitulate features of a minimal synthetic cell and are by definition larger than 1 μm in diameter, although in practice, most methods produce 10- to 100-μm-sized GUVs.^13−15^ Note that there is no precise definition of the exact size ranges for SUVs and LUVs.^16−19^ However, by most definitions, unilamellar liposomes larger than 200 nm and up to 1000 nm are greater than SUVs and smaller than GUVs. Therefore, vesicles in this size range can be considered as LUVs. SUVs up to 200 nm in diameter can serve to represent minimal virus particles or synthetic small EVs.^14,15,20,21^ A variety of production methods are available for the production of both GUVs and SUVs. In the case of SUVs and LUVs up to 250 nm, extrusion and sonication are the most common production methods.^22^ For GUV formation, the most common methods are gentle or gel-assisted hydration,^23^ electroformation,^24^ jetting,^25^ as well as reverse emulsion methods,^26,27^ including continuous droplet interface crossing encapsulation (cDICE)^28^ and charge-mediated droplet-stabilized technologies.^29−31^ Several reviews have covered their applications, advantages, and limitations.^13,24,32−34^ In stark contrast and despite the variety and importance of natural compartments in the size range of 200–1000 nm, the literature on methods for the controlled assembly of large unilamellar vesicles (LUVs) in this size range is scarce. Many reports on LUV formation methods date back to the 70s and 80s and most of these describe the formation of SUVs and LUVs up to 250 nm in diameter.^19,35−37^ These approaches most commonly rely on detergent removal, injection of organic solvent with phospholipids into an aqueous phase, or cyclic pH changes in a suspension of charged phospholipids. In publications that describe the production of LUVs larger than 250 nm, a large proportion of the obtained vesicles are in fact coproduced SUVs.^38,39^ The development of the reverse phase evaporation method advanced the production of vesicles between 200 and 1000 nm. However, multilamellarity remains a problem that cannot be solved without limiting the size of the vesicles by extrusion.^40^ More recently, a supercritical reverse phase evaporation method seems to have solved the issue of multilamellarity for large LUVs, but it requires a very complex workflow and specialized equipment.^41^ SUV fusion induced by freeze–thawing is an easier approach to LUV production. However, this method suffers from a broad size distribution of the produced vesicles including many SUVs and again, multilamellarity.^42−44^ Consequently, there is still an unmet need for well-characterized techniques for efficient and high-purity LUV formation between sizes of 200–1000 nm, ideally using widely available tools and a simple workflow. In this work, we use the term ‘purity’ to describe the absence of vesicles in undesired size ranges (i.e., below 200 and above 1000 nm in diameter).

Therefore, for LUV formation we adapted a droplet stabilization technology that was previously developed for charge-mediated GUV formation by microfluidics^31^ as well as shaking and vortexing.^29^ The method consists of the following steps: (1) SUV formation; (2) creation of an SUV-loaded, surfactant-stabilized water-in-oil emulsion; (3) charge-mediated attraction and fusion of SUVs at the inner droplet periphery, in other words, the emergence of one large LUV that is stabilized by a droplet shell (dsLUV) through fusion of the SUVs at the droplet periphery; and (4) release of the vesicle from the surfactant shell and surrounding oil phase into aqueous buffer.

By the implementation and optimization of an emulsifying device,^45^ essentially a high-rate stirrer that generates very high and reproducible shear forces, we achieved the formation of vesicles below 1 μm in diameter. We have systematically characterized the influence of different chemophysical parameters on the concentration and size of the resulting vesicles. In particular, we tested the effect of emulsification conditions (rotation speed, duration, and incubation time), lipid and Mg^2+^ concentration in the aqueous phase and surfactant concentration in the oil phase. Consequently, we have derived an optimal protocol for the simple and efficient production of high-purity LUVs specifically in the size range of 200–1000 nm, with a focus on negatively charged vesicles between 400 and 600 nm. Importantly, the method relies on equipment that is present in many laboratories and on commercially available materials.^46^ Additionally, we assessed the influence of the production method on the lipid composition and surface charge of the obtained vesicles. The outcomes of this research can serve as essential guiding tools for controlled LUV formation, which will be highly relevant for the future construction of synthetic cell organelles.

## Results and Discussion

### Optimization of Surfactant and Mg^2+^ Ion Concentrations for Efficient dsLUV Formation

**Figure 1:**
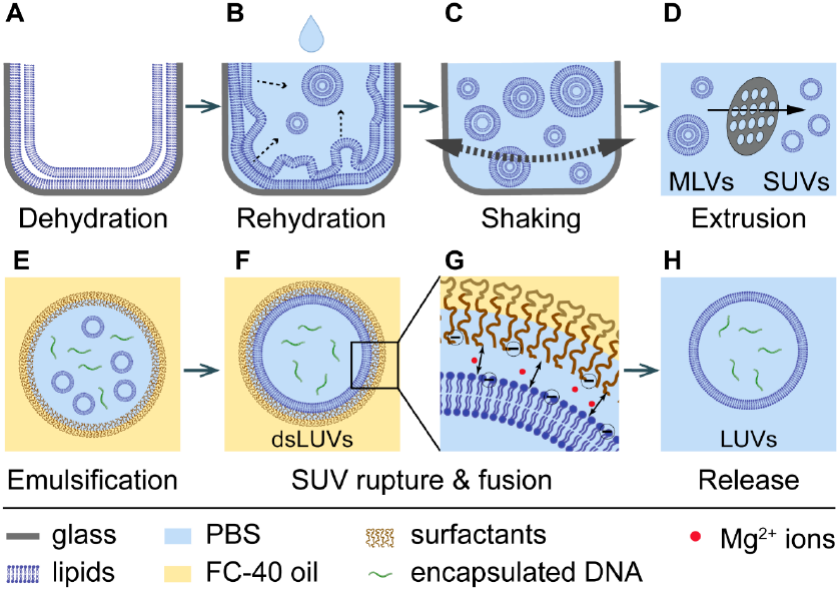
Schematic representation of the LUV production process. First, multilamellar vesicle (MLV) production is performed by drying (A) and rehydrating (B) a thin lipid film, followed by shaking (C). The MLVs are then converted into SUVs by extrusion (D). Emulsification creates SUV-loaded, surfactant-stabilized water-in-oil droplets (E), at the periphery of which SUVs are ruptured and fuse into a large vesicle due to charge-mediated interactions (F, G). Subsequently, vesicles are released into an aqueous buffer by a destabilizing surfactant (H).

In this study, for LUV formation, we adapted and optimized the charge-mediated, emulsion-stabilized vesicle assembly approach that has been previously developed for GUV assembly.^29^ Our method consists of four sequential steps: (1) SUVs are prepared by extrusion (Figure 1A–D) and combined with Mg^2+^ ions in an aqueous buffer. At this step, if desired, hydrophilic cargo to be encapsulated, can be added, e.g., DNA oligos, soluble dyes, etc. (2) The SUVs containing aqueous phase is added to the oil phase that contains a mixture of neutral, polyethylene glycol–perfluoropolyether (PEG–PFPE) block copolymer surfactant,^31^ and negatively charged PFPE–carboxylic acid (Krytox) surfactant (for more details see Materials and methods section). We used FC-40 oil, which has a high viscosity (4.1 cP). (3) The emulsifier is applied to generate a water-in-oil emulsion (Figure 1E). The size of the obtained droplets depends strongly on the strength of the mechanical shearing with higher shear rates leading to smaller droplets. During the emulsification process, both surfactants assemble at the droplet periphery in a competitive manner. The neutral surfactants mainly ensure droplet stability and the negatively charged surfactants attract Mg^2+^ ions from the SUV-containing buffer, which in turn, promotes negatively charged SUVs to fuse into a large vesicle at the droplet periphery (Figure 1F,G). In case of neutral or positively charged SUVs, fusion can be achieved by using a high concentration of KCl instead, albeit with lower yield.^29,31^ (4) Following dsLUVs formation, addition of a shorter, de-emulsifying surfactant allows for the destabilization of the surfactant shell and leads to LUV release into simultaneously added aqueous buffer (Figure 1H).^29,31^

**Figure 2:**
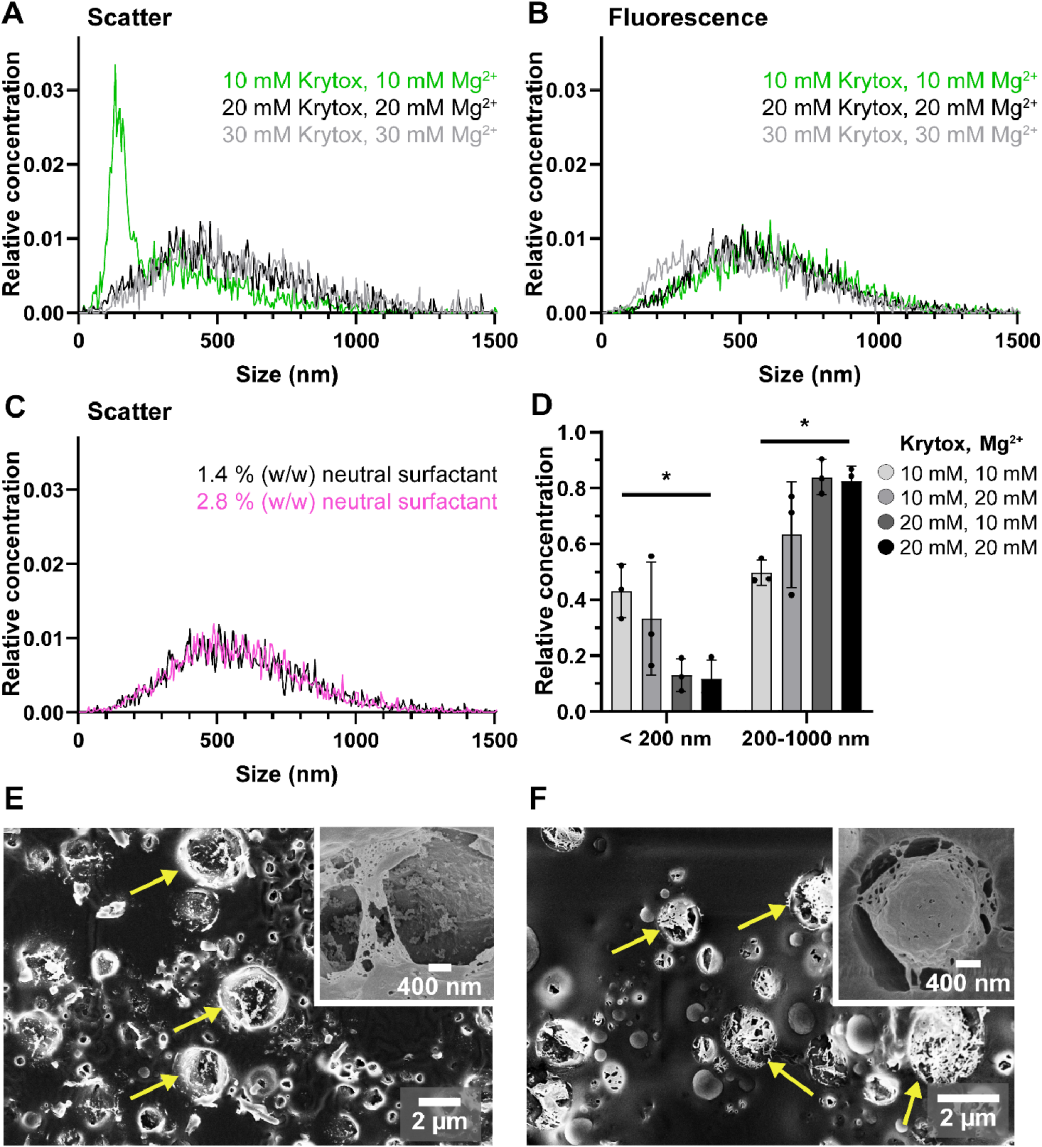
SUV fusion into LUVs can be improved by raising Krytox and Mg^2+^concentrations. (A) Relative size distribution of released vesicles produced with different surfactant and Mg^2+^ concentrations. The average of three independent replicates is shown for each condition. (B, C) Relative size distribution of the subset of released vesicles containing fluorescently tagged DNA. The average of three independent replicates is shown for each condition. (D) Relative concentrations of released vesicles below 200 nm in size and between 200 and 1000 nm in size, depending on Krytox and Mg^2+^ concentrations. Ordinary one-way ANOVA, * *p* < 0.05, error bars show SD for *n* = 3 independent replicates. (E, F) Cryo-SEM micrographs of dsLUVs before release. The dsLUVs were produced with either 10 mM Krytox and 10 mM Mg^2+^ (E) or 20 mM Krytox and 20 mM Mg^2+^. (F) Arrows highlight examples of the concentration-dependent coverage of the droplets with a lipid bilayer.

To produce LUVs between 200 and 1000 nm instead of GUVs, we optimized the use of an emulsifier (rotation speed and duration). The first step was to adjust the concentration of Krytox and Mg^2+^ ions based on previous GUV studies. There, the optimized experimental conditions consisted of 1.5 mM lipids and 10 mM Mg^2+^ ions in the aqueous phase and 10 mM Krytox surfactant and 1.4% (w/w) neutral fluorosurfactant in the oil phase and vortexing generated the emulsion.^29^ To create dsLUVs, we decided to increase the lipid concentration to 5.4 mM in order to compensate for the fact that the total surface area of all droplets increases when they are smaller in size. Moreover, we used the emulsifier at the highest speeds (speed 5 and 6, 18 000, and 28 800 rpm, respectively) for 30 and 180 s, each with the goal of creating the smallest possible droplets (Figure S1). Following the release of the vesicles to physiological conditions, nanoparticle tracking analysis (NTA) measurements revealed a very small median diameter of 130–150 nm for the obtained vesicles in most conditions (Figure S1). This size correlates well with the dimensions of the SUVs used for emulsification. Therefore, we decided to test whether the implemented conditions were at all sufficient to achieve the required charge-mediated SUV attraction and fusion at the droplets’ inner interface. Toward this end, we added Atto488-labeled DNA oligo molecules (22 nucleotides in length) to the SUV-containing aqueous phase before the emulsification process. As negatively charged DNA generally does not cross membranes and free DNA in solution was not detected by NTA (data not shown), all detected fluorescent particles must stem from SUV rupture and fusion process. Figure 2A,B (green curves) shows the size distribution of the released vesicles, as measured by scatter and fluorescence detection. In scatter mode (Figure 2A) there is a large concentration peak below 200 nm, which is absent in fluorescent mode. Hence, these small vesicles do not contain DNA and most likely represent unfused SUVs. These results confirm that charge-mediated SUV attraction and fusion are not efficient under the above-described conditions.

To enhance charge-mediated SUV attraction and fusion at the droplets’ inner interface, we increased the equimolar concentrations of Krytox and Mg^2+^ ions from 10 to 30 mM while all other experimental conditions were kept the same. NTA measurements revealed similar size distributions in scatter and 488 nm fluorescence modes for the vesicles produced with 20 mM or higher Krytox and Mg^2+^ concentrations (Figure 2A,B). Moreover, no scattering peaks below 200 nm were observed. This indicates a reduced number of unfused SUVs in the final LUV suspension.

As the neutral fluorosurfactant concentration plays an important role in the prevention of droplet coalescence after emulsification, we also tested the effect of increasing the neutral fluorosurfactant concentration from 1.4% (w/w) to 2.8% (w/w) but did not find this to influence size distribution (Figure 2C). We concluded that 1.4% (w/w) neutral fluorosurfactant is sufficient to stabilize droplets for LUV formation and used this concentration for all further experiments. On a side note, the ability of our method to encapsulate small nucleic acids or other hydrophilic cargo inside vesicles is advantageous for possible future biomedical applications. As an example of other cargo, we show the NTA detection of Calcein-loaded LUVs (see Figure S2F).

For further optimization of the LUV formation process, we tested different nonequimolar combinations of Krytox and Mg^2+^ (Figure 2D). Based on the DNA encapsulation experiments, we strove to minimize the fraction of vesicles smaller than 200 nm. The absolute concentration of 200- to 1000-nm-sized vesicles is in the range of 3.4 × 10^10^ to 5.3 × 10^10^ vesicles/mL and does not differ significantly between the tested conditions (see Figure S2B). As can be observed in Figure 2D, an increase in Krytox and Mg^2+^ concentrations led to a significant decrease in the relative fraction of undesired SUVs < 200 nm, whereas the fraction of LUVs in the size range of 200–1000 nm increased significantly to 83 ± 5% (mean ± SD relative vesicle concentration) at a 20 mM equimolar concentration of Krytox and Mg^2+^. As the quality of NTA measurements decreases when measuring vesicles above 1000 nm in size, we assessed the vesicle suspensions by confocal microscopy and confirmed that the number of vesicles larger than 1000 nm is negligible when produced under optimal conditions (Figure S2C,D).

Cryo-SEM measurements were performed for the high-resolution observation of dsLUV formation under different equimolar Krytox and Mg^2+^ concentrations. Freeze fracturing and sublimation of the dsLUVs allowed us to analyze the formation of the lipid bilayer on the droplet’s inner periphery. Figure 2E,F shows representative cryo-SEM micrographs of the freeze-fractured droplets obtained with low (10 mM) and high (20 mM) equimolar concentrations of Krytox and Mg^2+^, respectively. Analysis of the fractured droplets produced with higher concentrations revealed a higher and more continuous coverage of the inner droplet periphery by a fused lipid bilayer (lighter areas, highlighted by arrows). Note, neither this layer nor anything similar to it was observed when droplets were produced under the same conditions but without the encapsulated SUVs (Figure S2F). In contrast to droplets produced with high Krytox and Mg^2+^ concentration, only unfused SUVs or small disrupted patches of the lipid bilayer were observed in droplets produced with lower Krytox and Mg^2+^ concentrations (Figure 2E). This correlates with our observation that some formation of LUVs between 200 and 1000 nm also occurs with 10 mM each Krytox and Mg^2+^, but less efficiently in comparison to dsLUVs produced with higher Krytox and Mg^2+^ concentrations. Consequently, we decided to use equimolar 20 mM Krytox and Mg^2+^ concentrations for all further experiments to attain the best possible vesicle purity in the desired size range.

### Influence of Physical Emulsification Parameters on Final LUV Size and Concentration

**Figure 3:**
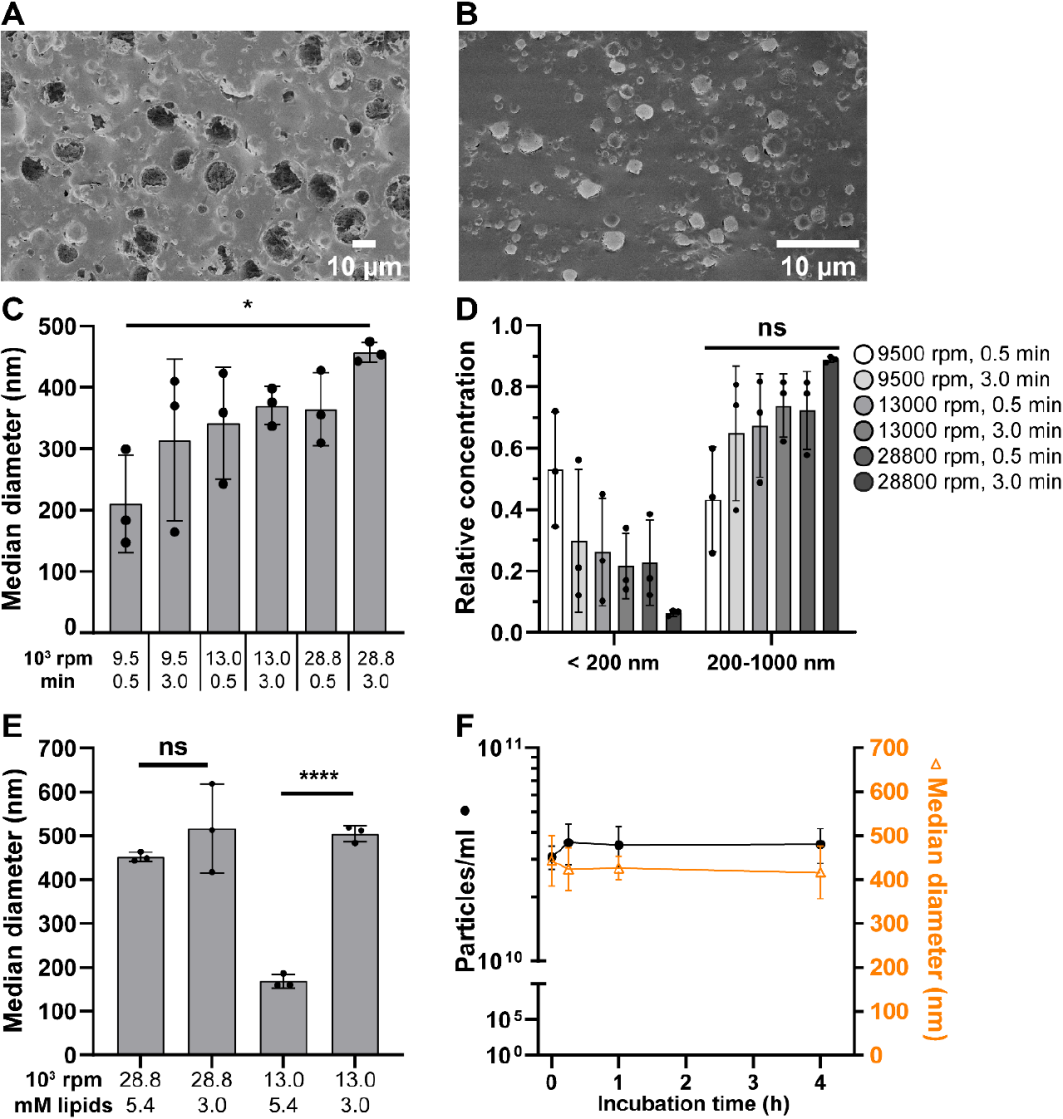
Influence of physical emulsification parameters on vesicle size and concentration. (A, B) Cryo-SEM micrographs of dsLUVs before release, produced with optimal Krytox and Mg^2+^ concentrations and either emulsification speed of 9500 rpm for 30 s (A) or 28 800 rpm for 3 min (B). (C) Median diameter of all released vesicles produced with optimal Krytox and Mg^2+^ concentrations depending on emulsification speed and time. Ordinary one-way ANOVA, * *p* = 0.046. (D) Relative concentrations of released vesicles below 200 nm and between 200 and 1000 nm in size in samples from (C). Ordinary one-way ANOVA, ns: nonsignificant (*p* = 0.07 for <200 nm and *p* = 0.05 for 200–1000 nm). (E) Median diameter of released vesicles produced with optimal Krytox and Mg^2+^ concentrations depending on lipid concentration and emulsification speed. Welch-ANOVA and Dunnett’s T3 multiple comparison, **** *p* < 0.0001. (F) Median diameter and concentration of released vesicles produced with optimal Krytox and Mg^2+^ concentrations and emulsification at 28 800 rpm for 3 min, depending on the duration of incubation between emulsification and release. All points in the figure represent independent replicates. ns: not statistically significant.

Following the optimization of the chemical parameters, we set out to characterize the influence of the physical parameters of the emulsification process, namely, speed and duration, on the size and concentration of the produced vesicles. As a greater emulsification speed creates higher shear forces, we assumed that it would lead to smaller dsLUVs, resulting in the release of smaller LUVs. To estimate the range of the resulting droplet dimensions, we produced two emulsions using very different conditions and analyzed the results by cryo-SEM. Figure 3A,B shows representative cryo-SEM micrographs of the emulsions produced with 9500 rpm for 30 s and 28 800 rpm for 180 s, respectively. In line with our expectations, much larger droplets of up to 10–15 μm were obtained employing the slower emulsification speed in comparison to droplets of up to 2 μm in size using the fastest emulsification speed.

Surprisingly, despite having implemented the optimized Krytox and Mg^2+^ concentrations, NTA analysis of the released vesicles revealed the opposite trend: an increase in median vesicle diameter from low to high speed and longer emulsification duration (Figure 3C). Moreover, only in the case of the highest emulsification speed and duration was it possible to achieve a very high purity of LUVs between 200 and 1000 nm, specifically 89 ± 1% (mean ± SD) relative concentration and only 6 ± 1% (mean ± SD) relative concentration of smaller vesicles (Figure 3D). In the other conditions, the relatively high concentration of lipids used in the aqueous phase (5.4 mM) might cause high numbers of unfused SUVs in the case of bigger droplets, as they have less surface area relative to volume. During the release and enrichment steps, breakage of the encapsulating vesicle can occur, which releases unfused SUVs in large numbers.

To establish whether that is the case, we first assessed again the cryo-SEM measurements on droplets produced at 9500 rpm (speed 2) for 30 s as well as droplets produced at 28 800 rpm (speed 6) for 180 s and compared these. As expected, SUV-like structures could be observed in the lumen of large droplets (Figure S3C), but not in smaller droplets (Figure S3D). In a next step, we prepared vesicles through emulsification either at 13 000 rpm (speed 4) or 28 800 rpm (speed 6) for 180 s and tested whether reducing the lipid concentration influences the occurrence of small, likely unfused, vesicles. As can be observed in Figure 3E, at 13 000 rpm, the median diameter of the released vesicles was significantly larger at lower lipid concentrations: 168 ± 15 nm obtained with 5.4 mM lipids compared to 504 ± 19 nm obtained with only 3.0 mM lipids. In contrast, at 28 800 rpm and a lipid concentration of 5.4 mM, the median diameter of the released vesicles was 452 ± 10 nm. Decreasing the lipid concentration to 3.0 mM did not significantly change the median vesicle diameter (516 ± 101 nm) at this speed but instead led to higher variation between the replicates and a reduced vesicle concentration (Figure S3E). This may be due to insufficient coverage of the droplet periphery by the lipids, thus, leading to incomplete vesicle formation and increased instability. The possibility of excess SUVs inside the droplets also led us to investigate by cryoTEM whether the LUVs formed with 5.4 mM lipids are unilamellar, or if the superfluous SUVs might form additional lamellae. We found that most LUVs were unilamellar as desired (Figure S3F). These findings indicate that by fine-tuning the emulsification conditions and lipid concentration, it is possible to achieve very precise control over the vesicle size.

One should take into consideration that during vesicle release and enrichment, the vesicle size distribution can be affected by several physicochemical factors.^29,31^ The change in size could be attributed to size-dependent release efficiency, osmotic pressure, and size-dependent mechanical instability of the vesicles. For instance, larger vesicles are likely less mechanically stable during the release process, pipetting, and centrifugation. Additionally, they might be pelleted and removed during the first centrifugation step, together with residual oil droplets. In addition to size-dependent instabilities, an osmotic pressure gradient between the solutions inside and outside of the vesicles can lead to either vesicle deflation or swelling.^29,47^ In this study, we did not match the LUVs’ inner buffer osmolarity to the osmolarity of the releasing buffer. Matching the osmolarity of the encapsulated and the release buffers can minimize osmotic effects on size and potentially further improve the vesicle yield.

While we have achieved very efficient and reproducible LUV production in the desired size range of 200–1000 nm, we recognize that this range is relatively broad, and a narrower size distribution of LUVs might be desired for specific applications. Therefore, we have included an exemplary experiment in which we narrowed down the size distribution of the LUVs to maximum 500 nm by postproduction filtration in a mini-extruder with a 400 nm filter membrane. Figure S3G and H, shows the narrower size distribution of the filtered LUVs and the concentration of the LUVs before and after filtering, indicating only acceptable material losses. Note, to obtain LUVs with a narrower size distribution implementation of filters with other pore sizes or other purification procedures such as size exclusion chromatography could be applied as well. It is important to mention here that immediate extrusion of the MLVs through 400 nm filters generates vesicles with the median diameter of 165 ± 1 nm (Figure S4). Moreover, the resulting vesicles are most likely still multilamellar due to the large pore size of 400 nm.^48^

We initially tried deriving the necessary incubation time after emulsification and before release from the literature by adding the time for SUV diffusion to the droplet periphery and the time for rupture and fusion. The diffusion coefficients of 100–200 nm liposomes are in the range of approximately 10^–12^ m^2^/s based on the Stokes–Einstein equation.^49^ Even if such a liposome travels the longest possible distance inside a 500 nm droplet before encountering the interface, this diffusion process takes only milliseconds and is thus negligible compared to the time required for rupture and fusion. The kinetics of SUV rupture and fusion at the inner periphery of a droplet are unknown, but quartz crystal microbalance with dissipation monitoring (QCM-D) studies have reported values for supported lipid bilayer (SLB) formation on solid surfaces, such as silica and mica. Generally, attachment of SUVs to the surface is already observed after 1–2 min, but the time required for rupture and fusion into an SLB varies largely, overall leading to SLB formation times ranging between 1.3 min to almost 1 h, depending on vesicle lipid composition, presence of ions in the buffer, and the surface material.^50,51^ As it is difficult to make predictions for our conditions based on these studies, we performed experiments to compare the influence of four different incubation times (0, 15, and 60 min and our standard incubation time of 240 min) on the final vesicle size and concentration, as measured by NTA (Figure 3F). We did not find any significant difference in either the median size or vesicle concentration. This indicates that dsLUV formation at the droplet periphery happens fast, at the latest within 15 min after emulsification. A similar observation has been made before for charge-mediated dsGUV formation.^29^ However, note that, although not statistically significant, the LUV concentration might be slightly lower and the LUV size slightly larger after 0 min incubation. In conclusion, it is possible either to proceed within a few minutes for a faster protocol or to store the emulsion for a few hours before release. We have summarized the optimized protocol for our LUV formation as a short step-by-step protocol in the Supporting Information, page 6.

### Alternative Lipid Compositions and Surfactants

We assessed the capability of the developed technology to form LUVs with different lipid compositions. In one composition, we increased DOPG content to 30% and reduced DOPC content to 69%. Additionally, we tested a more complex and physiologically relevant composition that mimics the lipid content of natural extracellular vesicles produced by mesenchymal human stem cells (MSC EVs).^52^ We found that there is no significant difference in the median LUV diameter depending on the DOPG content (Figure S6A). LUVs produced with 30% DOPG were 448 ± 19 nm in comparison to 457 ± 16 nm for 15% DOPG (*p* = 0.93, one-way ANOVA with Dunnett’s multiple comparisons was used for this and all following comparisons). Regarding the vesicle concentration (Figure S6B), we found that the method is slightly more efficient for the formation of LUVs containing 30% DOPG (1.0 × 10^11^ ± 1.3 × 10^10^ particles/mL) in comparison to vesicles containing 15% DOPG (4.7 × 10^10^ ± 2.7 × 10^9^ particles/mL, *p* = 0.0003). Higher concentration could be attributed to more efficient charge-mediated attraction and rupturing of SUVs containing 30% DOPG on the inner droplet interface due to their higher negative charge. The obtained median diameter and the concentration of the LUVs consisting of lipids mimicking the MSC EVs were 565 ± 54 nm (*p* = 0.015, compared to 15% DOPG, one-way ANOVA with Dunnett’s multiple comparisons) and 1.8 × 10^10^ ± 2.0 × 10^9^ particles/mL. Overall, these results demonstrate that the developed method for LUV formation can be adapted to different lipid compositions. Note, for neutral or positively charged lipid compositions, the use of KCl instead of MgCl_2_ might be required.^29,31^

Furthermore, we tested whether a different neutral surfactant can also be used. We tested the FluoSurf-O by Emulseo, which is a neutral fluorosurfactant of the formula PFPE-*b*-PPO–PEO–PPO-*b*-PFPE with a molecular weight between 7 and 13 kDa, and compared the results to our previous results using the PEG–PFPE-based neutral surfactant by Ran Biotechnologies. The comparison is shown in Figure S7. We did not find a significant difference between the surfactants, neither in median diameter of the produced vesicles (457 ± 19 nm for PPO–PFPE-based compared to 457 ± 16 nm for PEG–PFPO, *p* = 0.99, unpaired *t* test) nor in the vesicle concentration achieved (6.9 × 10^10^ ± 1.6 × 10^10^ particles/mL for PPO–PFPE-based, compared to 4.7 × 10^10^ ± 2.7 × 10^9^ particles/mL for PEG–PFPE-based, *p* = 0.08, unpaired *t* test), demonstrating the versatility of our method.

### Changes in Lipid Composition and Surface Charge During LUV Production

**Figure 4:**
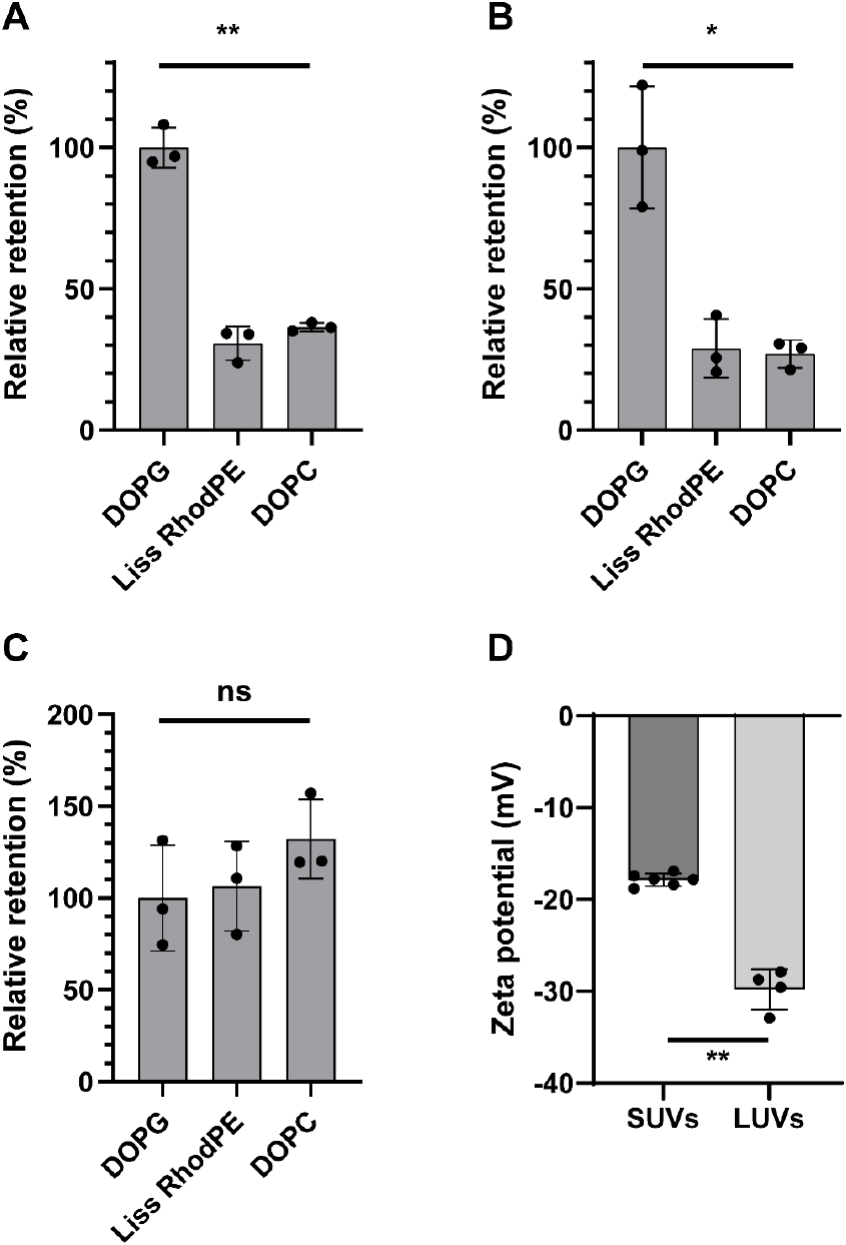
Mass spectrometric analysis of lipid retention throughout the LUV production process and zeta potential of the obtained LUVs. Lipid retention in LUVs compared to initial mixture in chloroform (A) or to SUVs (B) as well as lipid retention in SUVs compared to initial mixture in chloroform (C), shown relative to DOPG. Welch ANOVA: **p = 0.002 for A, *p = 0.03 for B, ns: not statistically significant, *p* = 0.36 for (C). (D) Surface charge of the vesicles as characterized by zeta potential measurements in 1× DPBS. Unpaired *t* test with Welch’s correction, **p = 0.001. All points in the figure represent independent replicates.

Slightly negatively charged LUVs are particularly interesting for potential future biomedical applications. Therefore, we used a lipid composition of 84% DOPC, 15% DOPG, and 1% Liss RhodPE for all experiments unless stated otherwise. We used mass spectrometry in order to assess whether the lipid composition is preserved during the LUV production process. We prepared LUVs using 5.4 mM lipids, 20 mM equimolar Mg^2+^ and Krytox, 1.4% (w/w) neutral fluorosurfactant, and emulsification at 28 800 rpm for 3 min. Note that we performed only a relative quantification by mass spectrometry, meaning that we did not derive absolute lipid compositions. Instead, we determined the relative retention of each lipid species from the initial chloroform mixture to LUVs. Our results suggest that there are significant changes in lipid composition (Figure 4). Compared to the initial lipid mixture in chloroform and relative to the retention of DOPG, only 31 ± 6% (mean ± SD) of Liss RhodPE and 36 ± 2% of DOPC were retained in LUVs (Figure 4A). In order to understand which step causes these changes, we also compared the lipid mixtures of LUVs and SUVs (Figure 4B). The results were very similar: 29 ± 10% and 27 ± 5% relative retention of Liss RhodPE and DOPC, compared to DOPG, respectively. In line with this, we did not find a significant difference in the lipid retention between the lipids from the initial chloroform mixture to SUVs (Figure 4C).

The changes in lipid composition that occurred during the charge-mediated formation of LUVs from SUVs or during the release process were also reflected in a higher negative charge of the LUVs (−29.8 ± 2.2 mV) in comparison with the SUVs (−17.9 ± 0.7 mV). This higher negative charge can be attributed to a higher retention of negatively charged DOPG lipids^53^ and a lower retention of neutral DOPC and Liss RhodPE lipids.^53,54^ We assume that a potential reason for neutral lipid losses can be attributed to their partial partitioning into the oil phase. Supporting our assumption, we detected a fluorescent signal in the oil phase after the formation of dsLUVs and release that indicates the presence of Liss Rhod PE in oil. One could speculate that the neutral charge of Liss Rhod PE and DOPC increases their lipophilicity compared to that of the negatively charged DOPG and thus their partial partitioning into the oil phase. Therefore, to compensate over the potential partial loss of neutral lipids, one could plan a slightly higher concentration of these lipids in SUVs composition.

Depending on the desired application of the LUVs, it has to be decided on a case-by-case basis whether this change in lipid composition and surface charge is acceptable, for example, if it is outweighed by the advantages of the method, such as high purity, efficiency, and simplicity.

## Summary and Conclusion

In this work, we adapted an emulsion-based, charge-mediated GUV formation protocol for the formation of negatively charged LUVs between 200 and 1000 nm in size, with a median diameter of 400–600 nm. Using cryo-SEM and nanoparticle tracking analysis, we characterized the influence of different biochemical and biophysical parameters on LUV formation, their size, and concentration. We also shed light on changes in the lipid composition and surface charge of the vesicles by mass spectrometry and zeta potential measurements, respectively. Our systematic study has allowed us to identify the ideal parameters for efficient, high-purity production of LUVs between 200 and 1000 nm in size using a simple protocol relying on widely accessible tools and reagents. The generated LUVs have high potential for future applications in synthetic biology and possible biomedical applications, mimicking natural large EVs or subcellular organelles. The designed method for charge-mediated LUV formation is not limited to a certain lipid composition. However, careful examination of the net charge of the lipid composition must be performed in order to adjust the buffer and surfactants composition. It must be considered individually which LUV properties are required, mainly whether changes in lipid composition as demonstrated here are acceptable, and certain parameters may need optimization to specifically suit the application. We have provided an extensive framework to guide researchers on this, detailing which parameters to consider and which outcomes to expect. Additional parameters, which were outside the scope of this study, to be considered in the future include utilizing oils with different viscosities and emulsifying devices with different geometries.

## Methods

### Vesicle Production

The lipids 1,2-dioleoyl-*sn*-glycero-3-phosphocholine (DOPC), 1,2-dioleoyl-*sn*-glycero-3-phospho-(1′-rac-glycerol) (sodium salt) (DOPG), 1,2-dioleoyl-*sn*-glycero-3-phosphoethanolamine-N-(lissamine rhodamine B sulfonyl) (ammonium salt) (Liss RhodPE), 1,2-dioleoyl-*sn*-glycero-3-phospho-l-serine (sodium salt) (DOPS), ovine cholesterol, 1,2-dioleoyl-*sn*-glycero-3-phosphoethanolamine (DOPE), N-nervonoyl-D-erythro-sphingosylphosphorylcholine (SM), 1,2-dioleoyl-*sn*-glycero-3-[(*N*-(5-amino-1-carboxypentyl)iminodiacetic acid)succinyl] (nickel salt) (DGS-NTA(Ni)), 1,2-dioleoyl-*sn*-glycero-3-phospho-(1′-myo-inositol) (ammonium salt) (DOPI), N-palmitoyl-D-erythro-sphingosine (ceramide), and 1,2-dioleoyl-*sn*-glycerol (DAG) were obtained from Avanti Polar lipids. SUVs and LUVs were produced as previously described by Macher et al.^46^ using a molar lipid ratio of 84% DOPC, 15% DOPG, and 1% Liss Rhod PE unless specified otherwise. The lipid composition of MSC EV LUVs consisted of: 42.6% DOPC, 23.9% DOPE, 10.9% DOPI, 7.6% SM, 4.3% DOPS, 4.3% ceramide, 2.2% DAG, 2.2% cholesterol, and 2.0% DGS-NTA(Ni). In brief, lipids dissolved in chloroform were mixed and desiccated. The resulting lipid film was incubated with 1× DPBS to a total lipid concentration of 6 mM for 10 min at room temperature. The sample was vortexed for 5 min to create multilamellar vesicles (MLVs). The MLV suspension was extruded an uneven number of times, at least 11, according to the manufacturer’s instructions using a MiniExtruder (Avanti Polar Lipids) though a membrane with 100 nm pore diameter. For data in Figure S4, a 400 nm pore size filter was used where noted. SUVs were combined with MgCl_2_ and, where applicable, 10 μM Atto488-labeled ssDNA oligo or 1 mM Calcein; the suspension was adjusted to 200 μL with 1× DPBS. This phase was placed on top of a 400 μL FC-40 oil phase containing the noted concentrations of Krytox 157 FSH (Krytox) and neutral 008-fluorosurfactant (Ran Biotechnology) in a 2 mL reaction tube. The neutral PEG–PFPE surfactant most likely consists of a mixture of 4 kDa diblock and 6 kDa triblock copolymers;^31^ the Krytox is a PFPE carboxylic acid of 7.0–7.5 kDa. For comparison to a different neutral surfactant, we used FluoSurf-O (Emulseo) at a concentration of 1.4% (w/w) instead of the neutral 008-fluorosurfactant. This surfactant consists of PFPE-*b-*PPO–PEO–PPO-*b*-PFPE and has a molecular weight between 7 and 13 kDa. The mixture was emulsified using an IKA T 10 basic ULTRA-TURRAX device equipped with the S10N-8G rotor/stator combination at an emulsification speed of 6 (28 800 rpm) for 3 min unless specified otherwise. Corresponding rpm values for the speed levels used in this study are presented in Table 1 as obtained from the manufacturer. As the emulsifier creates high shear forces and, thus, potential local heating, we tested whether cooling the emulsion in an ice water bath during emulsification alters the size and concentration of the released LUVs. However, we did not find any significant difference, neither in size nor concentration (Figure S5), so all further experiments were conducted without cooling. The emulsion was incubated at 4 °C for at least 3.5 h before release, unless specified otherwise. The vesicles were released from the oil phase by addition of 400 μL of 1× DPBS and 400 μL of de-emulsifying surfactant, 1H,1H,2H,2H-fluor-1-octanol, and overnight incubation at room temperature. The aqueous phase was filled up with 1× DPBS to reduce losses, transferred to a fresh 1.5 mL tube, and centrifuged at 21  300*g* at room temperature for 30 s to remove potential oil droplets. The supernatant was centrifuged under the same conditions for 1 h to pellet down the LUVs. The supernatant was removed, and the pellet carefully resuspended in 100 μL of 1× DPBS. The vesicles were stored at +4 °C to +8 °C until further analyses were performed on the same or following day. In one exemplary experiment, the produced 100 μL of LUV suspension were diluted with 900 μL 1× DPBS and then once very slowly pushed through a mini-extruder (as described above) with a membrane filter of 400 nm pore size until 250 μL were left in the injecting syringe. The filtered LUVs were collected on the other side of the extruder.

**Table 1:** Speed Levels of the IKA T 10 Basic ULTRA-TURRAX®

speed level	approximate rpm
2	9500
4	13 000
5	18 000
6	28 800

### Nanoparticle Tracking Analysis (NTA)

NTA was carried out on a ZetaView F-NTA Quatt instrument (ParticleMetrix) equipped with a custom set of bandpass emission filters. LUVs were diluted in 1× DPBS to achieve a concentration of approximately 100–200 particles per frame. The NTA settings were optimized to detect both SUVs and LUVs as follows: 24 °C, 11 positions, 1 cycle, highest quality, sensitivity 80, shutter 150, frame rate 15. A minimum area of 10, maximum area of 10 000, minimal brightness of 30, and trace length of 30 were used as postacquisition parameters. Measurements were carried out using the 488 nm laser and no emission filter for the scatter mode. The 488 nm laser and 525/50 nm emission filter were used to detect signals from encapsulated fluorescent DNA and Calcein. For the DNA measurements, low bleach mode was used and the following settings were changed compared to scatter measurements: sensitivity was adjusted to 90, shutter to 50 and minimal brightness to 25. For Calcein detection, the same mode was used except with sensitivity 70 and shutter 200. A minimum of 500 traces were analyzed for each measurement.

### Zeta Potential Measurements

Zeta potential measurements were carried out in zeta cuvettes on the dynamic light scattering device NanoSight ZS (Malvern Panalytical, Germany). Vesicles were diluted by 40–100× (depending on the concentration) in 1× DPBS and measured after 3–5 min equilibration time, at 25 °C and 173° backscatter. The measurement parameters attenuator and number of runs were set automatically by the device.

### Cryo-SEM

For assessing dsLUVs by cryo-SEM, emulsions were incubated at least 3.5 h after emulsification before being processed for cryo-SEM. A Zeiss Ultra 55 field-emission electron microscope (FE-SEM) was used for image acquisition (Zeiss SMT, Germany). Top-view cryo-SEM imaging was performed under low temperature conditions (−115 ± 5 °C). Low acceleration voltages of 2 kV were used due to the low conductivity of the investigated samples. Signals were detected by the in-lens detector. The emulsion droplet solution (3 μL) was dropped on 0.8 mm diameter gold specimen carriers assembled on a freeze fracture holder and immersed immediately in liquid nitrogen. After vitrification in liquid nitrogen, the droplets were transferred to a Leica EM BAF060 (Leica Microsystems) preparation device via an evacuated liquid-nitrogen-cooled shuttle VCT 100 (Leica Microsystems). For freeze-fracture cryo observations the droplets were fractured in the 10^–6^ mbar vacuum chamber at −160 °C. After fracturing, the stage was heated to −90 °C and kept in the vacuum chamber for 40 min in order to allow water in the fractured droplets to sublimate. To perform cryo-SEM the samples were transferred immediately to the SEM chamber via an evacuated liquid nitrogen cooled shuttle VCT 100.

### Cryo-TEM

Cryo-grids were glow discharged (45 mA, 90s). 3 μL of sample was applied to either C-Flat 1.2/1.3 or 2/1 girds. The grids were blotted for 3s with a blot force of −1 and plunge-frozen with a Vitrobot Mark IV (Thermo Fischer).

The frozen samples were imaged with a Titan Krios (Thermo Fischer) microscope operated at 300 kV. Images were acquired with EPU utilizing a Falcon III direct electron detector (Thermo Fischer) at a magnified pixel size of 2.94 Å per pixel. The accumulated total dose per image did not exceed 29e/Å per image.

### Confocal Microscopy

Vesicles were imaged on a Zeiss LSM800 laser scanning confocal microscope equipped with Plan-Apochromat 20× air and a 63× oil immersion objective. Vesicles were imaged on glass slides passivated with 1% (w/v) BSA for 10 min at room temperature.

### Mass Spectrometry

The samples for mass spectrometry analysis of the vesicle lipids were produced in three independent replicates, and each of them was measured in duplicates. Each replicate experiment compares the initial lipid mixture in chloroform, SUV suspension in 1× DPBS, and LUV suspension in 1× DPBS. Lipids in chloroform, SUV suspensions, and LUV suspensions in 1× DPBS were diluted in acetonitrile by a factor of 40 000, 20 000, and 2000, respectively, to achieve similar lipid concentration levels. The samples were sonicated for 5 min after initial dilution. As quantitative internal standard (IS), the EquiSPLASH LIPIDOMIX mixture (Avanti) was added to each sample to achieve a final concentration of 0.05 μg/mL (per lipid). This internal standard enables us to correct for variations originating from sample preparation and measurement procedure as well as nonlinear concentration effects, which can differ between lipids. Note that the mixture does not contain an internal standard for Liss RhodPE.

A ratiometric liquid chromatography tandem mass spectrometry (LC–MS/MS) was carried out using a Sciex QTRAP 4500 triple quadrupole mass spectrometer hyphenated with a Shimadzu Nexera UPLC (HILIC setup) equipped with a Waters XBridge Amide column (3.5 μm, 4.6 mm × 150 mm). The column was kept at 35 °C and the on-column injection volume was 5 μL. Elution was performed with solvent 1 (50/50 (v/v) ACN/H_2_O with 10 mM ammonium acetate, pH 8) and solvent 2 (95/5 (v/v) ACN/H_2_O with 10 mM ammonium acetate), both of which were produced from LC–MS or higher-grade reagents. The employed flow gradients and solvent concentrations are listed in Table 2. From 13 to 23 min an increased flow rate (up to 1 mL min^–1^) was utilized (and directed into the waste) to ensure efficient column cleaning and to prevent cross-contamination of lipids between samples. The Sciex Analyst 1.7.2 software was used to control the multireaction monitoring (MRM). The MSMS and electrospray ionization (ESI) source parameters of the MRM method are presented in Table 3. MSMS fragmentation patterns and MRM parameters were derived by syringe infusion of the lipid standards dissolved in a 50/50 (v/v) mixture of dichloromethane and methanol supplemented with 10 mM ammonium acetate to enable adduct formation.

**Table 2:** Gradient Information for the Hilic Separation of Lipids Using Solvent 1 (50/50 (v/v) ACN/H_2_O Supplemented with 10 mM Ammonium Acetate, Adjusted to pH 8) and Solvent 2 (95/5 (v/v) ACN/H_2_O Supplemented with 10 mM Ammonium Acetate) and a Waters Xbridge Amide Column (3.5 mM, 4.6 mM × 150 mM)

time (min)	parameter	value
0.01	solvent 2	99.9%
1.5	solvent 2	99.9%
4	solvent 2	94.0%
10.5	solvent 2	40.0%
11.5	solvent 2	0.0%
13	total flow	0.55 mL
13.5	solvent 2	0.0%
13.5	total flow	1.00 mL
19.1	solvent 2	0.0%
19.2	solvent 2	99.9%
22.5	total flow	1.00 mL
23	total flow	0.55 mL
24.5	stop	-

**Table 3:** Electrospray Ionization (ESI) Source and MS Parameters for the Ratiometric Analysis of Lipid and Steroid Components of Vesicles via LCMS (MRM Mode)

**ESI-positive**							
			LissRhodPE	DOPC	DOPC-d7	DOPG	DOPG-d7
curtain gas (psi)	35	Q1 (Da)	1301.605	786.528	753.600	792.440	759.500
collision gas (AU)	9	Q3 (Da)	682.000	184.000	184.000	603.600	570.600
source temp. (°C)	350	dwell (ms)	100.000	75.000	25.000	75.000	25.000
nebulizer gas (psi)	65	DP (V)	40.000	161.000	165.000	50.000	65.000
heater gas (psi)	70	CE (V)	67.000	39.000	39.000	25.000	33.000
ionization voltage (V)	5500	CXP (V)	24.000	14.000	13.000	16.000	15.000
entrance potential (V)	10						

**ESI-negative**							
			18:1 PC-OAc	18:1 PC-d7-OAc			
curtain gas (psi)	35	Q1 (Da)	844.500	811.500			
collision gas (AU)	9	Q3 (Da)	281.300	288.300			
source temp. (°C)	350	dwell (ms)	100.000	25.000			
nebulizer gas (psi)	65	DP (V)	–90.000	–115.000			
heater gas (psi)	70	CE (V)	–54.000	–50.000			
ionization voltage (V)	–4500	CXP (V)	–7.000	–11.000			
entrance potential (V)	–10						

The areas under the curve (AUC) for the investigated lipids were calculated using Sciex MultiQuant 3.0.2 software. The ratio of lipid/internal standard lipid (IS ratio) was calculated for each lipid and production step (chloroform mixture, SUVs, LUVs). For pairwise comparison between the production steps, the IS ratio of the earlier production step was used as a one-point calibrator and set to 100%. For example, the IS ratios of the LUVs were compared to the IS ratios of the initial chloroform mixture. The resulting values were averaged for technical duplicates. This average retention value of DOPG was then used to normalize the retention of Liss RhodPE and DOPC, resulting in the relative lipid retention values (%) that are shown in Figure 4.

## Supporting Information Available

Figure S1: Initial conditions led to inefficient LUV formation, Figure S2: Influence of chemical emulsification parameters on vesicles size and concentration, Figure S3: Influence of physical emulsification parameters on vesicle size and concentration, Figure S4: Comparison of vesicles from emulsification method and MLV extrusion through 400 nm pores, Figure S5: Effect of cooling during emulsification, Figure S6: Applicability of the developed LUV formation method for different lipid compositions, Figure S7: Implementation of different neutral commercially available surfactants for LUV formation. Summary of the optimized protocol for LUV formation (PDF)
