# Supplementary material for: An Efficient Method for the Production of High-Purity Bioinspired Large Unilamellar Vesicles
Source: ACS Synth Biol. 2024 Feb 29;13(3):781–91. doi: 10.1021/acssynbio.3c00540 (PMC10949243; doi:10.1021/acssynbio.3c00540)
Supplement: Supplementary file 1 — sb3c00540_si_002.pdf [file sb3c00540_si_002.pdf]

## Supporting Information

# An efficient method for the production of high-purity bioinspired large unilamellar vesicles

Meline Macher [a, b, c], Amelie Obermeier [a], Sebastian Fabritz [a], Massimo Kube[d], Hannah Kempf [a], Hendrik Dietz[b, d], Ilia Platzman* [a, c], Joachim P. Spatz* [a, b, c]

### Affiliations

[a] Max Planck Institute for Medical Research, Jahnstraße 29, 69121 Heidelberg, Germany

[b] Max Planck School Matter to Life, Jahnstraße 29, 69121 Heidelberg, Germany

[c] Institute of Molecular Systems Engineering and Advanced Materials, Im Neuenheimer Feld 225, 69120 Heidelberg, Germany

[d] Technical University of Munich, Am Coulombwall 4a, 85748 Garching, Germany

* Corresponding Authors: ilia.platzman@mr.mpg.de; spatz@mr.mpg.de

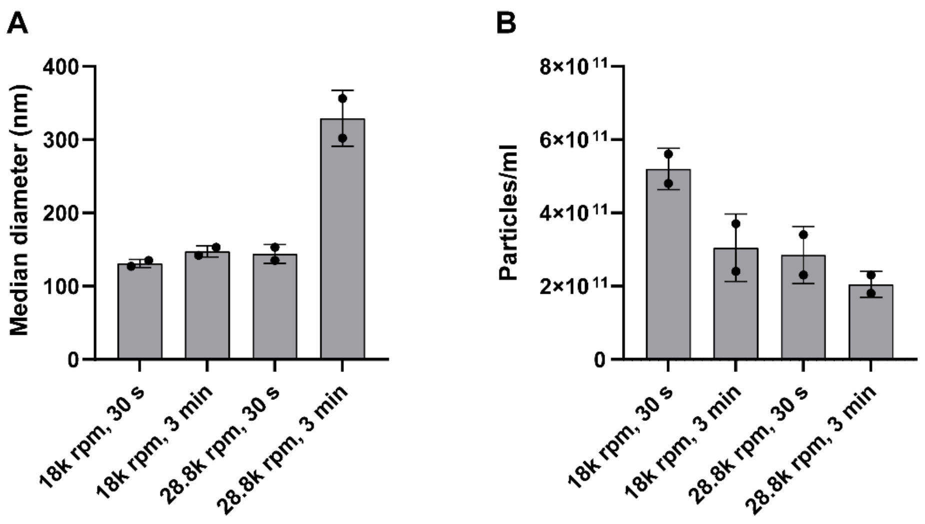


***Figure S1: Initial conditions led to inefficient LUV formation.*** *Median diameter (A) and vesicle concentration (B) as a result of emulsification with 5.4 mM lipids and 10 mM* $Mg^{2+}$ *in the aqueous phase and 10 mM Krytox and 1.4 % (wt/wt) commercial fluorosurfactant in FC-40 oil, at the indicated speeds and durations. n=2 independent replicates.*

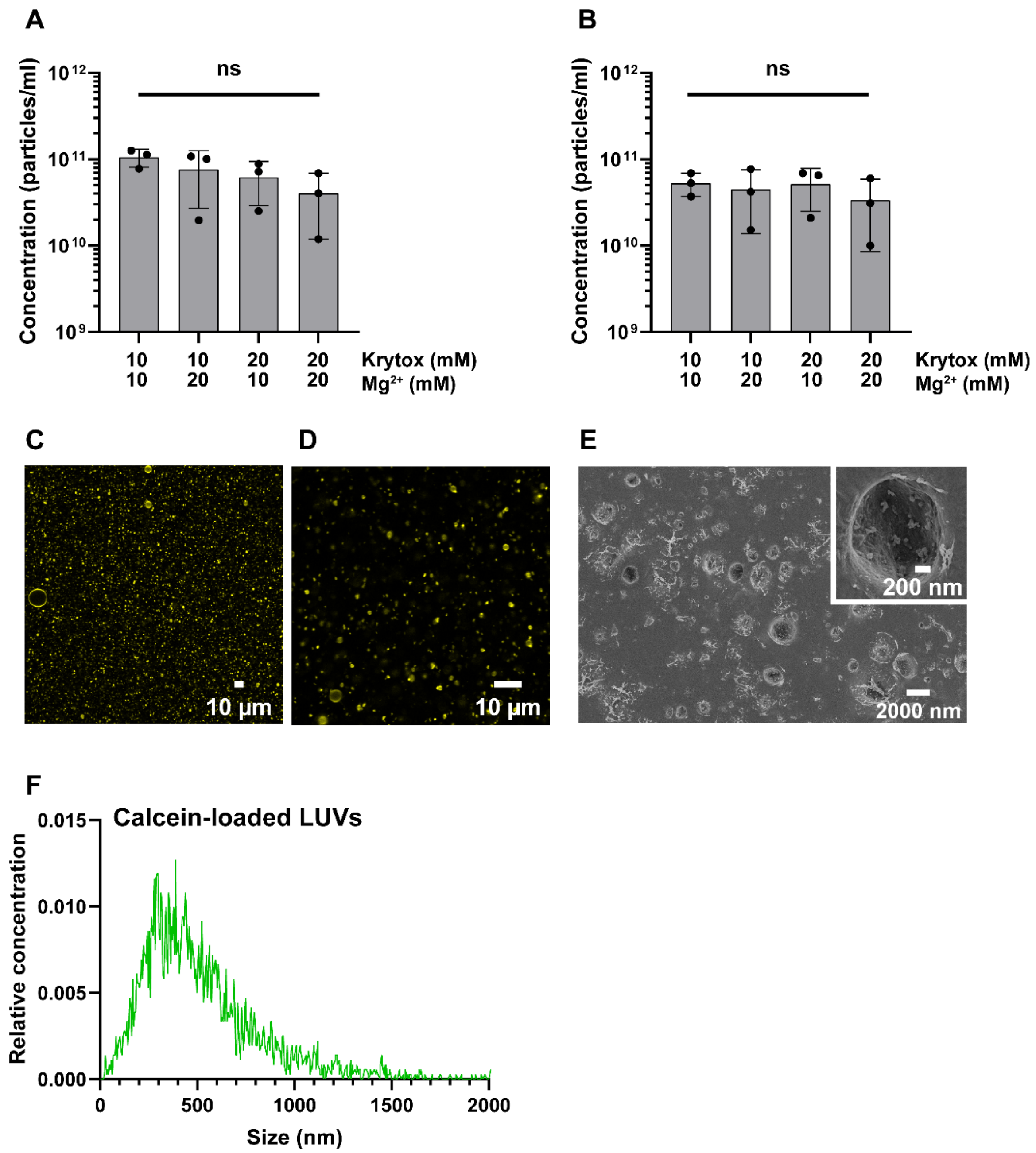


***Figure S2: Influence of chemical emulsification parameters on vesicle size and concentration.*** *Concentration of total released vesicles (A) and released LUVs between 200-1000 nm (B). Ordinary one-way ANOVA for both, ns: not statistically significant (p=0.22 for A and p=0.78 for B), n= 3 independent replicates. Confocal microscopy at 20x (C) and 63x (D) magnification of LUVs prepared with 20 mM Krytox, 20 mM $Mg^{2+}$, 1.4 % (wt/wt) commercial fluorosurfactant and emulsification at 28800 rpm for 3 min. (E) cryo-SEM micrographs of the water-in-oil emulsion prepared without lipids, otherwise using the same conditions as in C-D. (F) Size distribution of Calcein-loaded LUVs.*

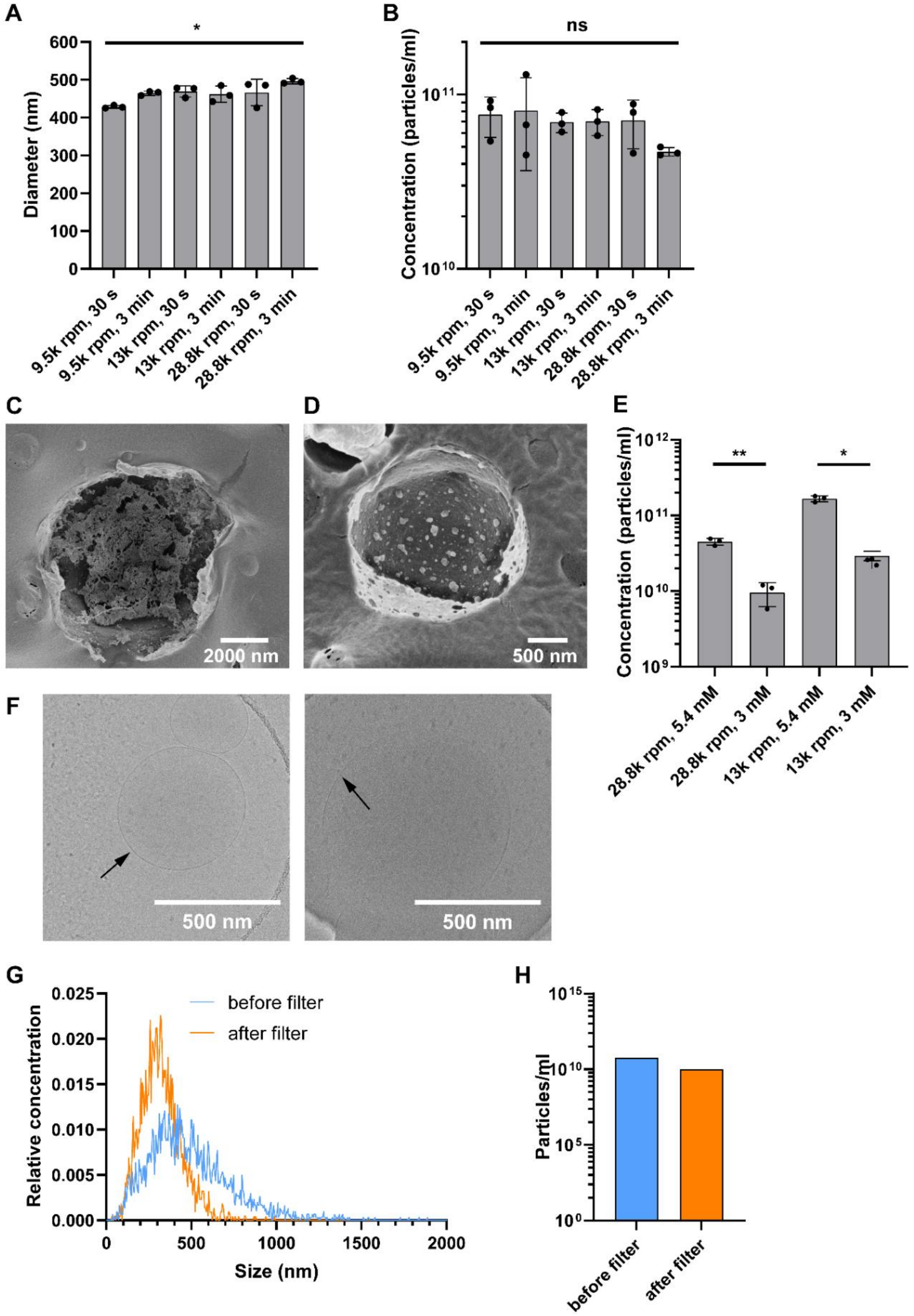


***Figure S3: Influence of physical emulsification parameters on vesicle size and concentration.*** *Median diameter (A) and concentration (B) of released LUVs in the desired size range of 200 nm-1000 nm. Both ordinary one-way ANOVA, *p=0.02 for A; ns: not statistically significant (p=0.55) for B. Cryo-SEM micrographs showing the contents of droplet-stabilized vesicles of different sizes produced with 5.4 mM lipids, either at 9500 rpm for 30 s (C) or at 28800 rpm for 3 min (D). Concentration of released vesicles (E) depending on lipid concentration and emulsification speed (for 3 min each). Welch ANOVA and Dunnett's T3 multiple comparisons, * p<0.05, ** p<0.005. All points in A, B and E represent independent experiments. (F) Representative cryo-TEM micrographs showing unilamellarity of the LUVs produced with the optimized protocol. Arrows point to the membrane. Size distribution (G) and absolute concentration (H) of LUVs before and after a single filtering step post-production through a 400 nm pore size extrusion device. Shown is an exemplary experiment.*

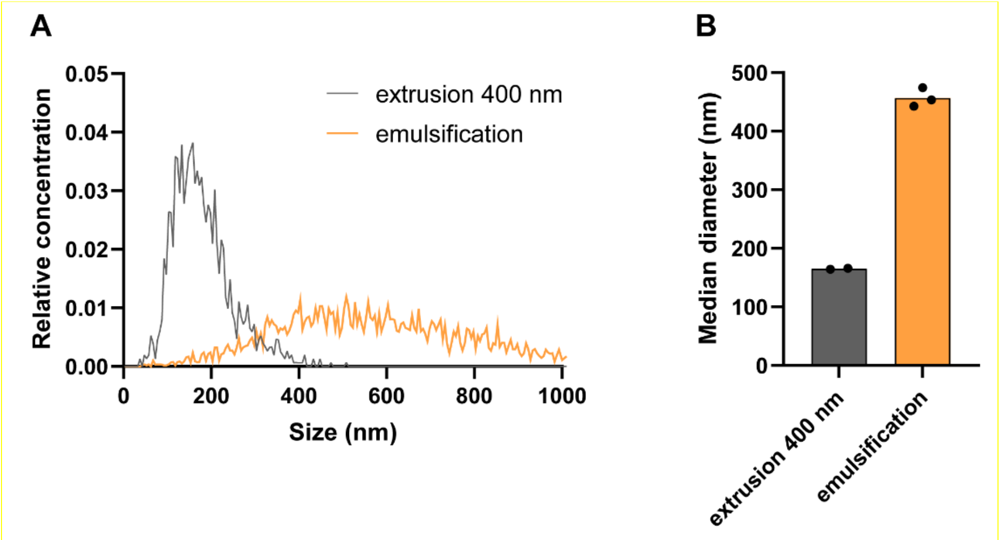


*Figure S4:* ***Comparison of vesicles from emulsification method and MLV extrusion through 400 nm pores.*** *Relative size distribution (A) and median diameter (B) of MLVs extruded through a 400 nm pore size filter (dark grey) and LUVs produced by emulsification (orange), as detected by NTA. Curves in A show the average of 2 independent replicates for extrusion and 3 independent replicates for emulsification. Points in B represent independent replicates.*

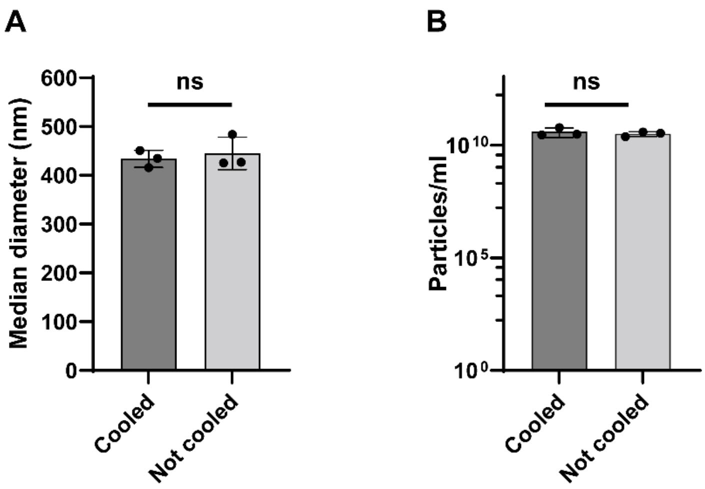


***Figure S5: Effect of cooling during emulsification.*** *Effect of cooling in an ice bath during emulsification on released vesicle size (F) and vesicle concentration (G). Unpaired t-test, p>0.5 for F and G, ns: not statistically significant. All points in the figure represent independent replicates.*

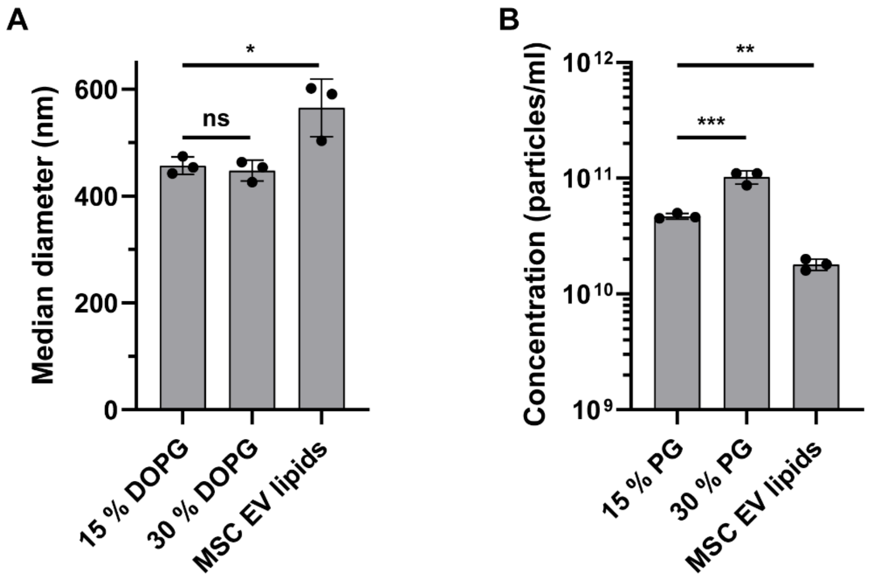


***Figure S6: Applicability of the developed LUV formation method for different lipid compositions.*** *Median diameter (A) and vesicle concentration (B) for the formation of LUVs consisting of two different lipid compositions in comparison to the standard one are presented. One-way ANOVA with Dunnett's multiple comparisons was performed. *p=0.015; **p=0.007; ***p=0.0003. All points in the figure represent independent replicates. ns: not significant.*

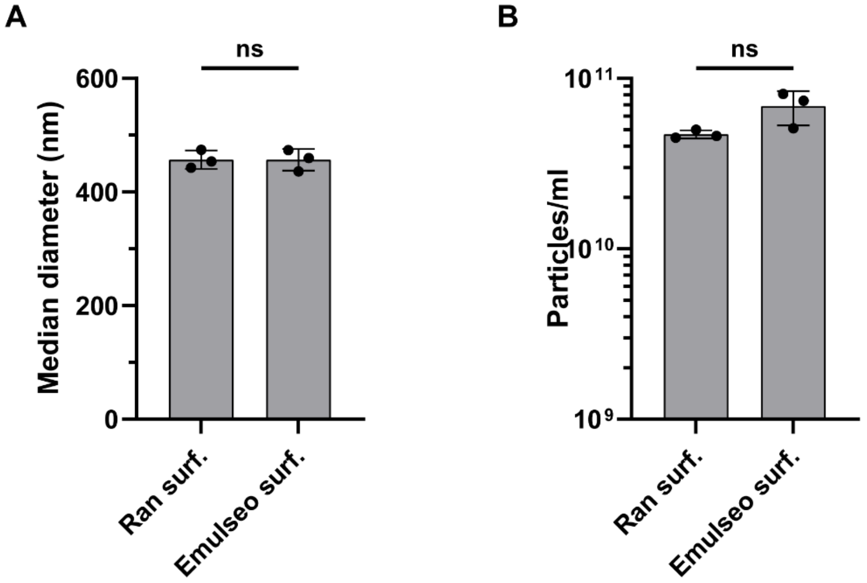


***Figure S7: Implementation of different neutral commercially available surfactants for LUV formation.*** *Median diameter (A) and vesicle concentration (B) when using different neutral fluorosurfactants. The data for Ran surfactant has been already presented in Fig. 3 and is shown as a comparison for the Emulseo surfactant. Unpaired, two-tailed t tests were performed. All points in the figure represent independent replicates. ns: not significant, surf.: surfactant.*

**Summary of the optimized protocol for LUV formation**

Carry out all procedures at room temperature unless otherwise specified. Use 0.22 µm-filtered 1x DPBS for all steps of the protocol. Similarly, filter FC-40 and use only the filtered oil.

SUV formation

1. Add the lipids dissolved in organic solvent such as chloroform into a glass vial according to the desired molar ratios and final aqueous volume (usually 200-1000 µl) and total lipid concentration (usually 6 mM).
2. Remove the solvent, e.g. by placing in a desiccator connected to a vacuum pump for at least 25 min.
3. Swell the lipid film by 10 -15 min incubation with the appropriate volume of 1x DPBS to achieve the total lipid concentration of 6 mM.
4. Shake the vial on a horizontal vortexer at 1000 rpm for at least 5 min to form multilamellar vesicles (MLVs).
5. Extrude MLVs according to the instructions of the extrusion kit through a polycarbonate membrane with a pore size of 100 nm.

ds-LUV formation

1. Place 400 µl of oil phase (**1.4% w/w neutral fluorosurfactant and 20 mM Krytox™ in FC-40**) in a 2.0 ml reaction tube.
2. Pre-mix 200 µl of aqueous phase (**5.4 mM total lipids, 20 mM $MgCl_2$ in 1x DPBS**) and then immediately place on top of the oil phase.
3. Emulsify at **28.8k rpm** (speed 6 on the IKA T 10 basic ULTRA-TURRAX®) **for 3 min**.
4. The emulsion can be stored for several hours at 4 °C. For optimal efficiency, we recommend an **incubation of at least 15 min** before release.

LUV release and enrichment

1. Carefully add 400.0 µl of 1x DPBS, followed by 400.0 µl of de-emulsifying surfactant 1H,1H,2H,2H-perfluoro-octanol to the emulsion.
2. Gently rotate the tubes to help detaching and maximize the water/oil interface. Incubate at room temperature until upper and lower phase are mostly translucent, some agglomerates will most likely remain at the interface. Released LUVs are in the upper phase.
3. If you desire to minimize losses, gently fill the tube with 1x DPBS before transferring the upper phase into a fresh tube.
4. Centrifugation allows to then increase the concentration of the LUV suspension again. A first centrifugation at 21300 rcf for only 30 s is recommended to remove potentially transferred oil droplet, while a second centrifugation of the resulting supernatant at 21300 rcf for at least 1 h pellets the LUVs. Gently resuspend in 1x DPBS.
